# Breast cancer in Cape Verde: a 24-year retrospective study of clinical presentation, treatment and outcomes at Agostinho Neto University Hospital

**DOI:** 10.3332/ecancer.2025.1826

**Published:** 2025-01-16

**Authors:** Pamela Borges, Hirondina Borges Spencer, Stefani Furtado, Victor Costa, Carla Barbosa, Lúcio Lara Santos

**Affiliations:** 1 Molecular Biology Laboratory, Agostinho Neto University Hospital, Praia, Plateau PC-112, Cabo Verde; 2 Clinical Research and Innovation Center, Agostinho Neto University Hospital, Praia, Plateau PC-112, Cabo Verde; 3 Serviço de Oncologia, Agostinho Neto University Hospital, Praia, Plateau PC-112, Cabo Verde; 4 Serviço de Cirurgia, Agostinho Neto University Hospital, Praia, Plateau PC-112, Cabo Verde; 5 Serviço de Patologia, Agostinho Neto University Hospital, Praia, Plateau PC-112, Cabo Verde; 6 Experimental Pathology and Therapeutics Group, Research Center of IPO Porto (CI-IPOP)/RISE@CI-IPOP (Health Research Network), Portuguese Oncology Institute of Porto (IPO Porto)/Porto Comprehensive Cancer Center (Porto.CCC), Porto 4200-072, Portugal; 7 Surgical Oncology Department, Portuguese Institute of Oncology, Porto 4200-072, Portugal; a https://orcid.org/0000-0002-0521-5655

**Keywords:** breast cancer, Cape Verde, epidemiology, molecular subtypes, survival

## Abstract

**Background:**

Breast cancer is a significant health concern in Cape Verde, but comprehensive data on its presentation, management and outcomes are limited. This study aims to provide insights into breast cancer patterns in this island nation.

**Methods:**

We conducted a retrospective analysis of 586 breast cancer patients treated at Agostinho Neto University Hospital in Praia, Cape Verde, from January 2000 to May 2024. Data on demographics, clinical presentation, diagnostic methods, treatment modalities and survival outcomes were collected and analysed.

**Results:**

The study population comprised 578 (98.6%) females and 8 (1.4%) males. The mean age at diagnosis was 52.1 years (SD 13.6) for females and 70 years (SD 16.7) for males. Stage III was the most common presentation (39.4%). Invasive ductal carcinoma was the predominant histological type. Immunohistochemical analysis in 307 patients revealed 69.4% luminal, 26.1% triple-negative and 4.6% HER2-positive subtypes. Treatment primarily involved surgery combined with chemotherapy and/or hormone therapy, with 33.4% receiving radiotherapy. The median follow-up was 36.5 months (range: 1–298 months), and the median survival time was 137.1 months.

**Conclusion:**

This study reveals breast cancer patterns in Cape Verde that share similarities with other African nations, including younger age at diagnosis and higher rates of late-stage presentation compared to Western countries. However, encouraging trends in survival outcomes and diagnostic capabilities were observed. These findings highlight the need for improved early detection strategies and expanded access to comprehensive treatment modalities, particularly radiotherapy, in Cape Verde.

## Background

Breast cancer remains a significant global health concern, ranking as the most common cancer among women worldwide. According to the World Health Organisation, in 2020, there were an estimated 2.3 million new cases of breast cancer globally [1]. It is the leading cause of cancer-related deaths among women in many countries, particularly in low- and middle-income nations [2]. Breast cancer incidence rates vary significantly across regions, with higher rates in developed countries [3]. Mortality rates are disproportionately higher in developing countries due to late-stage diagnosis and limited access to treatment [4]. In Cape Verde, an archipelagic nation off the west coast of Africa with 583,233 inhabitants, breast cancer presents unique challenges and patterns that warrant investigation. While comprehensive data on breast cancer in Cape Verde is limited, according to the recent people-based cancer registry of Cape Verde, breast cancer is the most common cancer and the second cause of death in females. Incidence and mortality age-standardised rate (World) per 100,000 females, were 23.4 and 7.8, respectively [5]. While oncology care, organisational improvements and diagnostic and treatment capabilities were initially established in 2000, significant progress was achieved in 2010 through enhanced diagnostic resources. Further advancement occurred in the last 5 years through support for team training and the establishment of a patient transfer program to Portugal for radiotherapy treatments.

Agostinho Neto University Hospital (HUAN) in Praia, Cape Verde, serves as the national reference centre for oncology, providing comprehensive diagnostic services, surgical oncology and medical oncology facilities. It remains the country’s sole healthcare institution equipped to treat oncological diseases. Through a bilateral agreement with Portugal, radiation therapy services are provided to Cape Verdean patients. The hospital has recently enhanced its capabilities with the addition of immunohistochemistry facilities and a molecular cancer laboratory. Additionally, the institution has expanded its treatment options to include targeted oncological drugs and established a Biological Safety Cabinet for chemotherapy preparation.

This study aims to address the knowledge gap by providing a comprehensive analysis of breast cancer cases treated at HUAN over the past 24 years. The research offers valuable insights into the local presentation, clinical management and treatment outcomes of breast cancer in Cape Verde, while also examining the evolutionary changes in care delivery during this period.

## Methods

### Study design and setting

This retrospective study analysed data from 586 consecutive patients diagnosed with breast cancer and treated at HUAN in Praia, Cape Verde. The study period extended from (Jan 2000) to (May 2024). HUAN serves as the primary referral centre for cancer care in Cape Verde.

### Patient selection

All patients diagnosed with breast cancer admitted and treated at the hospital during the study period were included. The diagnosis was confirmed through clinical examination, imaging studies and cytological and histopathological analysis. Patients with incomplete medical records or those who did not receive breast cancer treatment were excluded.

### Data collection

Medical records of eligible patients were reviewed by trained research assistants using a standardised data extraction form. The following data were collected:

- Demographic information: age, sex and residence.
- Clinical presentation: TNM Classification System (which assesses tumour size [T], regional lymph node involvement [N] and presence or absence of distant metastasis [M]).
- Diagnostic methods: mammography, ultrasound and biopsy results.
- Histopathological features: tumour type, hormone receptor status and HER2 status.
- Treatment modalities: surgery, chemotherapy, radiotherapy, hormonal therapy and target therapy.
- Outcomes: overall survival according to stage, molecular profile, treatments and 5-year periods.

### Follow-up standard protocol

We implemented a standardised follow-up protocol at HUAN in 2000, comprising quarterly assessments (by medical oncologist) during the first 2 years post-treatment, semi-annual evaluations from years three through five and annual follow-up thereafter, with additional consultations provided when patients presented with new symptoms or concerns between scheduled visits. Each follow-up assessment comprised:

- A comprehensive clinical examination, including systematic evaluation of both the affected and contralateral breast and the assessment of lymph node basins for potential recurrence;
- Annual mammographic and ultrasonographic imaging, with increased frequency when clinically indicated;
- Monitoring of tumour marker CA 15–3: biannually for the first 3 years, then annually thereafter.

Protocol adherence and patient-provider communication were overseen by a designated nurse navigator. Non-compliant patients received telephone follow-up with subsequent appointment rescheduling. For deceased patients, both the date and cause of mortality were documented. The surveillance period extended from initial diagnosis until either the study conclusion date, patient mortality or loss to follow-up. Patients were classified as lost to follow-up following two consecutive missed appointments and three unsuccessful contact attempts.

### Ethical considerations

This study was conducted according to international standards for good clinical practices. It was approved by HUAN, the Cabo Verde National Committee for Data Protection *(Comité Nacional de Proteção de Dados de Cabo Verde* (approval numbers: 26/2021 and 181/2022)), the Cabo Verde National Ethics Committee for Health Research (*Comité Nacional de Ética para Pesquisa em Saúde de Cabo Verde* (approval numbers: 3/2021 and 61/2022)). Patient confidentiality was maintained throughout the study, and all data were anonymised before analysis.

### Statistical analysis

Data were analysed using (SPSS version 23). Descriptive statistics were used to summarise patient characteristics, clinical features and 5-year period characterisation. Kaplan-Meier survival analysis was performed to estimate overall survival rates. Cox proportional hazards regression was used to identify factors associated with survival outcomes. A *p*-value <0.05 was considered statistically significant.

### Study limitations

Limitations of this study include long-period analysis, its retrospective nature and the potential for missing data in medical records. To reduce possible biases, we divided patients by 5-year periods; however, the follow-up period in the last 5 years is short. Additionally, as a single-centre study, the results may not be fully representative of the entire Cape Verdean population.

## Results

**Figure 1.**
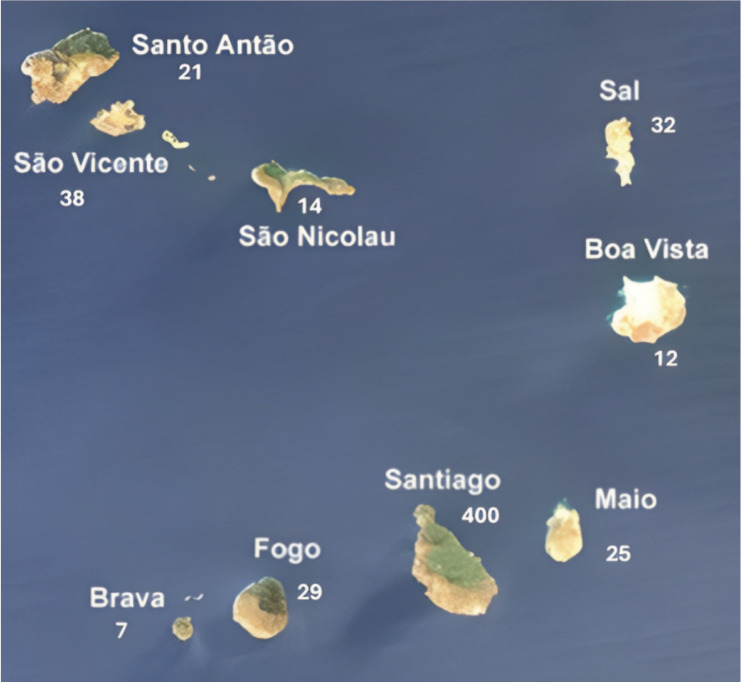
Number of cases per Island. In five cases, the origin was unknown and three were immigrants.

A total of 586 breast cancer patient records were analysed from the specified period at HUAN. Of these, 578 (98.6%) females and 8 (1.4%) males. The mean age at diagnosis was 52.1 years (SD 13.6) for females and 70 years (SD 16.7) for males. Most cases (68.3%) resided on the island of Santiago (Figure 1). The clinical, pathological and therapeutic characteristics of the series are condensed in Table 1. The diagnosis was primarily based on clinical examination, mammography and ultrasound in all patients. Histological confirmation was obtained in all cases. Invasive ductal carcinoma was the most frequent histological type observed. Immunohistochemical analysis in 307 patients revealed 69.4% luminal, 26.1% triple-negative and 4.6%

**Figure 2.**
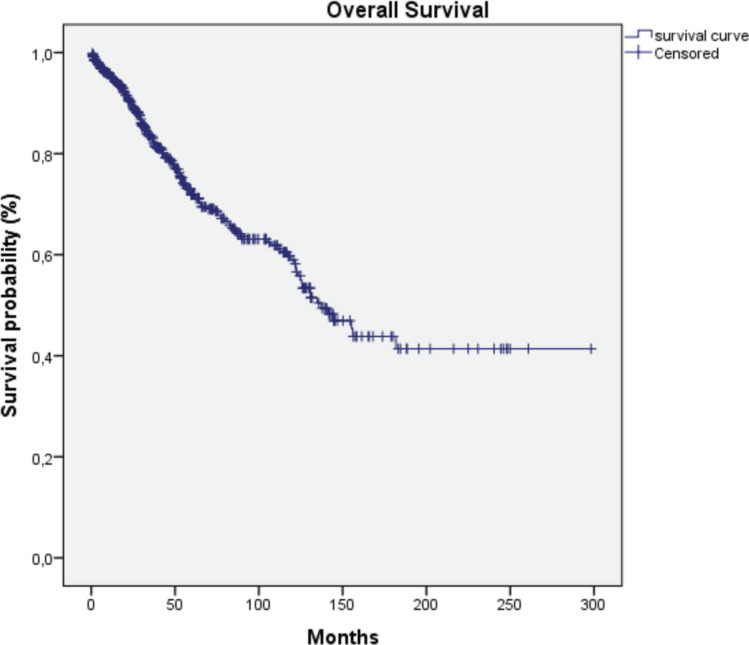
Overall survival curve of the series studied.

**Figure 3.**
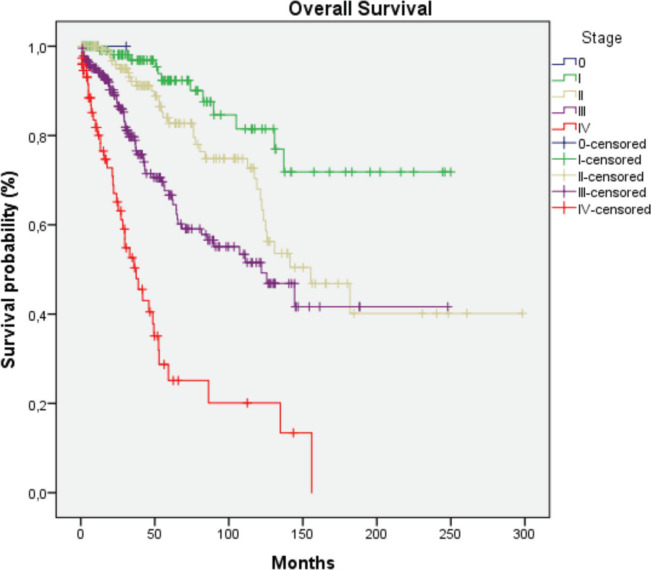
Survival curve according to the stage. Log Rank p < 0.001.

HER2-positive subtypes. Stage III was the most common presentation (39.4%). The most common treatment approach was surgery combined with chemotherapy and/or hormone therapy. Additionally, 196 patients (33.4%) received radiotherapy as part of their treatment regimen. The primary surgical approach was modified radical mastectomy, palliative mastectomy and breast-conserving surgery performed in selected cases. Therefore, few patients (*n* = 3) with cancer at the initial stages underwent breast-conservative surgery. Systemic treatment protocols included chemotherapy and hormonal therapy as primary modalities, while targeted therapy was administered to patients with HER2-positive disease. Twelve patients (2%) received the best supportive care only, likely due to advanced disease at presentation. The median follow-up time was 36.5 months (range: 1–298 months), and the median survival time was 137.1 months (Figure 2). We observed a consistent increase in the number of cases over the 5 years and at all stages (Table 2). Since there is no radiotherapy in Cape Verde, most patients are treated with surgery and systemic treatment. The agreement with Portugal for the evacuation of patients requiring radiotherapy enabled that from 2010 onwards there was an increase in patients benefiting from radiotherapy (Table 3). Standard radiotherapy was administered to patients following the established National Comprehensive Cancer Network protocol guidelines. We did not observe significant differences at 5 years in the overall survival of the 5-year period that can be compared (2005–2009, 2010–2014 and 2015–2019. Locally advanced and distantly metastasised tumours had significantly lower overall survival (Figure 3).

## Discussion

This study provides valuable insights into breast cancer trends and management in Cape Verde, offering a unique opportunity to compare the findings with both African and Western contexts. Furthermore, the research elucidates the evolution of oncological care for breast cancer patients in Cape Verde over time. The observed variations in breast cancer case volumes across the different regions of Cape Verde, as well as the fluctuations over the study period, are likely attributable to a confluence of factors. These include differences in population characteristics between the islands, such as variations in size, demographic profiles and health-seeking behaviours. Additionally, the uneven distribution and accessibility of cancer diagnosis and treatment resources throughout the archipelago likely played a role. Furthermore, gaps and inconsistencies in the reporting and registration of cancer cases within the national healthcare system may have contributed to the disparities observed.

Regarding demographics and clinical presentation, the mean age at diagnosis (52.1 years for women) in our study is similar to that reported in other African countries such as Nigeria (50 years) and Ghana (49 years), but lower than in Western countries like the United States (62 years) or the United Kingdom (60 years) [6–10]. This earlier onset in African populations may reflect differences in genetic factors, environmental exposures or population age structures [11].

The high proportion of stage III diagnoses (39.4%) aligns with trends seen across Africa. For instance, studies in Nigeria and Ghana report late-stage diagnoses in 70%–90% of cases [6–8]. This contrasts sharply with Western countries, where early stage diagnoses (stages I and II) predominate due to effective screening programs [9, 10].

The late presentation in Cape Verde, while concerning, is less severe than in some other African nations, possibly indicating better awareness or healthcare access.

The predominance of invasive ductal carcinoma is consistent with global patterns, including both African and Western populations. The distribution of molecular subtypes in our study (69.4% luminal, 26.1% triple-negative and 4.6% HER2-positive subtypes) is noteworthy. The high proportion of triple-negative breast cancers (TNBC) is similar to findings in other African countries like Ghana (82% luminal, 16% TNBC) and Nigeria (77% luminal, 16% TNBC). This contrasts with Western populations, where TNBC typically accounts for 10%–15% of cases [7, 8, 12, 13]. The high TNBC prevalence in African populations may have implications for prognosis and treatment strategies [14]. With respect to the low incidence of invasive lobular carcinoma in our study, the prevalence of lobular carcinoma in Sub-Saharan Africa is not extensively documented, with most studies focusing on other breast cancer subtypes. However, existing research indicates that invasive ductal carcinoma is the most common histological type, overshadowing lobular carcinoma [15]. The molecular subtypes identified in studies show a significant representation of aggressive forms, particularly TNBC and luminal A types, with lobular invasive carcinoma being less frequently reported [15].

The multimodal treatment approach in Cape Verde is commendable, aligning with international standards. However, the limited access to radiotherapy (33.4% of patients) is a concern shared across many African nations. In contrast, radiotherapy is more readily available in Western countries, with utilisation rates often exceeding 60% for breast cancer patients [16]. The median survival time of 144 months is encouraging and compares favorably with other African countries, nevertheless, several patients complete their treatment abroad, and this fact biases the data. For instance, studies in Nigeria and Ghana report 5-year survival rates of 56% and 49%, respectively [7, 8]. While this falls short of Western standards (5-year survival rates of 90% in the US and 85% in the UK), it suggests that Cape Verde is making significant strides in breast cancer management despite resource limitations. A comparison of the number of patients and therapeutic options over the 5-year periods studied reveals an increasing number of patients and an improvement in the therapies offered. However, we did not observe statistically significant differences in overall survival for each 5 years that could be studied. Notably, we observed overall (in the entire series studied) a significant increase in survival in the earliest stages, in patients treated with surgery, systemic treatment and radiotherapy. Patients with a luminal molecular profile have a better prognosis, as observed in other studies. The availability of immunohistochemistry and targeted treatments at HUAN represents progress that surpasses many other African nations. Most patients are from the island of Santiago, revealing that it is necessary to increase the oncology offer on other islands in Cape Verde, such as São Vicente. The need to send patients abroad for radiotherapy highlights ongoing infrastructure challenges common across the continent.

## Conclusion

This study reveals that breast cancer patterns in Cape Verde share similarities with other African nations, particularly in terms of younger age at diagnosis and higher rates of late-stage presentation compared to Western countries. However, Cape Verde shows some encouraging trends, such as better survival outcomes and advancing diagnostic capabilities, which position it as an intermediate case between typical African and Western scenarios. These findings underscore the need for tailored strategies in Cape Verde, focusing on earlier detection through improved screening programs and public awareness campaigns. Additionally, efforts to expand access to comprehensive treatment modalities, particularly radiotherapy, should be prioritised. Future research should investigate the genetic and environmental factors contributing to the high prevalence of TNBC in this population, including the occurrence of male patients and cases in individuals under 45 years of age. By addressing these challenges and building on existing strengths, Cape Verde has the potential to significantly improve breast cancer outcomes and serve as a model for other African nations facing similar healthcare challenges.

## Conflicts of interest

The authors declare that there is no conflicts of interest related to the publication of this article.

## Author contributions

This study was conceptualised, designed and written by Borges P and Santos LL. It was written by Santos LL. Acquisition of data was carried out by Borges P, Furtado S, Barbosa C, Costa V and Spencer HB. Analysis and interpretation of data were done by Santos LL and Borges PCC. All authors read and agreed to the final version of this manuscript.

## Figures and Tables

**Table 1.** Clinical, pathological and therapeutic characteristics of the series.

	Cases (*n*)	Percentage (%)
Gender Female Male	578 8	98.6 1.4%
Histhologic type DCIS Invasive ductal carcinoma Invasive lobular carcinoma Medullary carcinoma Papillary carcinoma Paget disease of breast (invasives) Cribiform invasive carcinoma Secretory carcinoma Epidermoid carcinoma Invasive breast cancer (NOS) Sarcoma	5 378 8 1 6 2 2 1 6 176 1	0.9 64.5 1.4 0.2 1 0.3 0.3 0.2 1 30 0.2
Molecular profile (only 307 cases) Luminal HER2 Triple negative	213 14 80	69.4 4.6 26.1
Decades 2000–2004 2005–2009 2010–2014 2015–2019 2020–2024	13 40 111 155 267	2.2 6.8 18.9 26.5 45.6
Stage 0 I II III IV	2 127 151 231 75	0.3 21.7 25.8 39.4 12.8
Treatment performed Surgery*+systemic treatment** Surgery*+systemic Treatment*+radiotherapy Systemic treatment** Surgery* Best supportive care Surgery*+Radiotherapy Systemic treatment**+radiotherapy Unknown	258 187 66 52 12 7 2 2	44 31.9 11.3 8.9 2 1.2 0.3 0.3

* Modified radical mastectomy, palliative mastectomy and breast-conserving surgery

** Chemotherapy and/or hormonotherapy

**Table 2.** Distribution of cases by 5-year period and stage (*x*^2^ *p* = 0.05).

5-year periods	Stage (cases)					Total
	0	I	II	III	IV	
2000–2004	0	3	8	2	0	13
2005–2009	0	8	16	11	5	40
2010–2014	1	25	31	44	10	111
2015–2019	0	39	40	53	23	155
2020–2024	1	52	56	121	37	267
Total	2	127	151	231	75	586

**Table 3.** Treatments performed in each 5-year period (number of cases).

5-year periods	Treatment performed								Total
	S+ ST+R	ST	ST+R	S+R	BSC	S	S+ST	U	
2000–2004	3	0	0	0	0	0	10	0	13
2005–2009	12	2	0	0	0	2	24	0	40
2010–2014	44	4	0	1	2	7	53	0	111
2015–2019	49	16	0	3	2	16	68	1	155
2020–2024	79	44	2	3	8	27	103	1	267
Total	187	66	2	7	12	52	258	2	586

**S+ST:** Surgery+systemic treatment; **S+ST+R:** Surgery+systemic treatment+radiotherapy; **ST:** Systemic treatment; **S:** Surgery; **BSC:** Best supportive care; **S+R:** Surgery+Radiotherapy; **ST+R:** Systemic treatment+radiotherapy and **U:** unknown
